# Manipulating Tyrosine Phosphorylation by Heterobifunctional Small Molecules

**DOI:** 10.1021/acscentsci.3c00836

**Published:** 2023-08-15

**Authors:** Xianke Meng, Jun Qi

**Affiliations:** Department of Cancer Biology, Dana-Farber Cancer Institute, Boston, Massachusetts 02215-5450, United States; Department of Medicine, Harvard Medical School, Boston, Massachusetts 02215, United States

**Figure 1:**
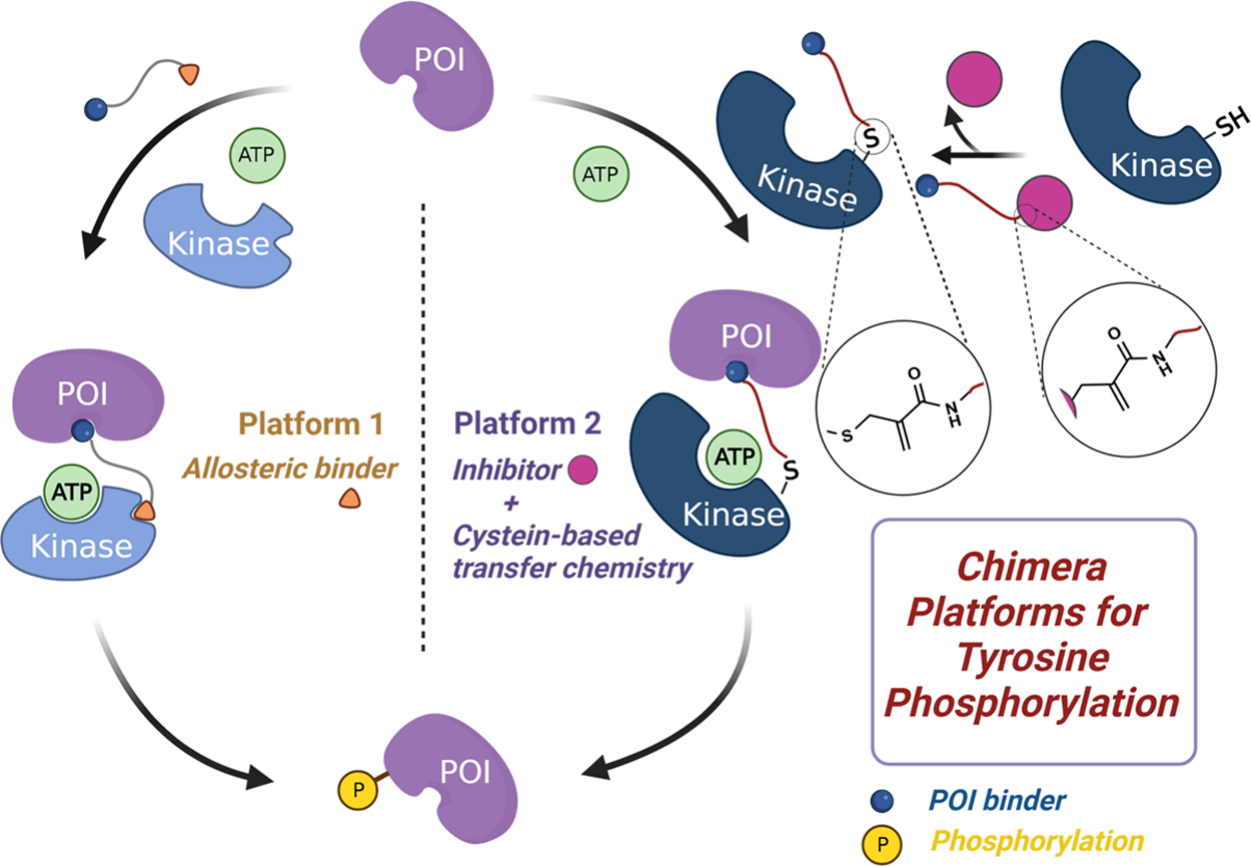
Chimera platforms for direct modulation of tyrosine phosphorylation.

Enzymatic functions are tightly regulated to maintain cellular survival in both normal and diseased settings. Pharmacological strategies that can directly exploit enzymes to modify specific proteins of interest (POI) would offer a powerful tool to control a protein’s function. Amit Choudhary and co-workers have been exploring a novel chimera strategy to pharmacologically direct phosphorylation onto a targeted protein. Protein phosphorylation is a prevalent post-translational modification (PTM) that plays a crucial role in numerous biological contexts. The Choudhary group previously reported a method using phosphorylation-inducing chimeric small molecules (PHICS) to recruit endogenous serine/threonine kinases to specific POIs, thus inducing phosphorylation.^1,2^ In this issue of *ACS Central Science*, Amit Choudhary and co-workers have broadened their approach using allosteric kinase binders and, additionally, built a second chemical biology platform that can convert enzymatic inhibitors into novel heterobifunctional small molecules (Figure 1).^3^

Recently, a variety of heterobifunctional small molecules have been developed to induce proximity of two or more proteins to trigger proximity-dependent modifications onto the POI. Proteolysis-targeting chimeric molecules (PROTACs) were first introduced in 2001 and are widely used throughout biomedical research to induce proteasomal degradation of target proteins, leading to new breakthroughs in drug discovery and chemical biology.^4^ More recently, the concept of bifunctional molecules has been applied to control and regulate transcription factor activity.^5^ With tyrosine phosphorylation (pTry) playing a significant role across the human proteome, the authors applied their previously described PHICS strategy to construct tyrosine PHICS. However, since PHICS need enzymes to maintain their activity, enzymatic kinase inhibitors cannot be directly utilized for kinase recruitment. To combat this, the authors first utilized noninhibitory binders of Abelson kinase (ABL) to design pTyr PHICS, which has allosteric binding sites available (Figure 1, platform 1).

Through integration of pocket probing, molecular docking, and a modified synthetic strategy, the authors successfully created a diverse library of ABL binders. As a proof-of-concept study, BRD4 was used as the target protein. The authors synthesized and evaluated their library of pTyr PHICS, which were comprised of ABL binders, a PEG linker, and (*S*)-JQ1, a BRD4 binder. Phosphorylation of BRD4 induced by ABL PHICS was observed, and the dihydropyrazole scaffold was identified to induce the greatest increase in pTyr. PHICS-induced BRD4:ABL ternary complex formation was confirmed biochemically. Further examination of BRD4 phosphorylation in 293T cells transfected with ABL-FLAG and BRD4-HA demonstrated that ternary complex formation is specific to ABL-PHICS. Additionally, PHICS exhibits catalytic turnover, as seen with PROTACs and previously reported Ser/Thr PHICS.^1,2^ Interestingly, ABL PHICS can phosphorylate signaling relevant tyrosine in diverse sequence environments on epidermal growth factor receptor (EGFR), a member of the receptor tyrosine kinases, further activating downstream EGFR signaling. Moreover, ABL PHICS containing a covalent binder (chloroalkane linker) can induce significant tyrosine phosphorylation on the target protein (HaloTag) in cells, indicating a favorable tolerance toward irreversible binding. These studies demonstrate that ABL can be recruited to diverse targets via ABL-PHICS to induce tyrosine phosphorylation and represents a major design strategy to develop novel PHICS.

While these findings are exciting on their own, the current PHICS strategy requires the usage of noninhibitory allosteric binders, which limit their advancement and development across all enzyme targets. To further expand the scope of PHICS, the authors built a second platform by utilizing abundantly available kinase inhibitors and cysteine-based transfer chemistry. They leveraged an inhibitor-directed release site-specific labeling strategy, enabling the installation of the POI binder to a near active-site cysteine residue on the target protein, which can then recruit the POI and achieve targeted protein phosphorylation (Figure 1, platform 2).^6,7^ By applying cysteine-based transfer chemistry, the potent ATP-competitive inhibitor can serve as a template to chemically transfer a covalent “allosteric binder” onto the kinase to achieve PHICS.

In a second proof-of-concept study, BRD4 PHICS were synthesized by connecting JQ1 to Bruton’s tyrosine kinase (BTK) inhibitor scaffolds through a cleavable methacrylamide linker. Formation of a ternary complex between BTK and BRD4, as well as the resulting phosphorylation of BRD4, was evaluated. The authors also confirmed the labeling of BTK and demonstrated the release of the BTK inhibitor scaffold from the ATP-binding pocket.^8^ Consistent with these findings, elevated levels of autophosphorylation in BTK were observed when treated with PHICS, surpassing the levels observed with the parent inhibitor. To demonstrate the generality of the strategy, the two platforms were combined, resulting in an ABL-BTK PHICS bifunctional molecule containing a covalent inhibitor of BTK (Ibrutinib). ABL-BTK PHICS was shown to effectively induce BTK phosphorylation in cells. Furthermore, the authors evaluated the effect of ABL-BTK PHICS on the viability of BTK-dependent and ibrutinib-resistant cancer cells (Mino and Raji cell lines).^9^ Remarkably, ABL-BTK PHICS demonstrated the ability to induce cell death in both cell lines, indicating its potential to overcome drug resistance.

Overall, the study by Amit Choudhary and co-workers not only demonstrated that PHICS can be utilized to target different types of kinases but also highlighted the utilization of cysteine transfer chemistry to broaden the scope of this approach. The success of the examples reported here takes advantage of the large number of inhibitors (both catalytic and allosteric) available for tyrosine kinases as well as the availability of crystal structures of small molecule inhibitors bound to protein. However, some tyrosine kinases lack structural information, thus making the design of PHICS challenging. Additionally, not all kinases may have reactive or surface exposed cysteines to implement this strategy. The selectivity of the POI phosphorylation sites is also of great importance to address for future development of PHICS. Future improvement of this strategy could benefit from the optimization of PHICS (e.g., selectivity, potency), conducting mechanistic studies to elucidate how PHICS induce cell death in drug-resistant cancer cells and investigating the feasibility of using PHICS in living animals. The current study can also be further expanded to the enzymes beyond the phosphorylation.^10^ As the majority of small molecule inhibitors target the enzymatic binding pocket, the extension of the PHICS strategy demonstrated here creates avenues for the application of protein manipulation to different enzymes and different modifications to answer a wider range of biological questions.
